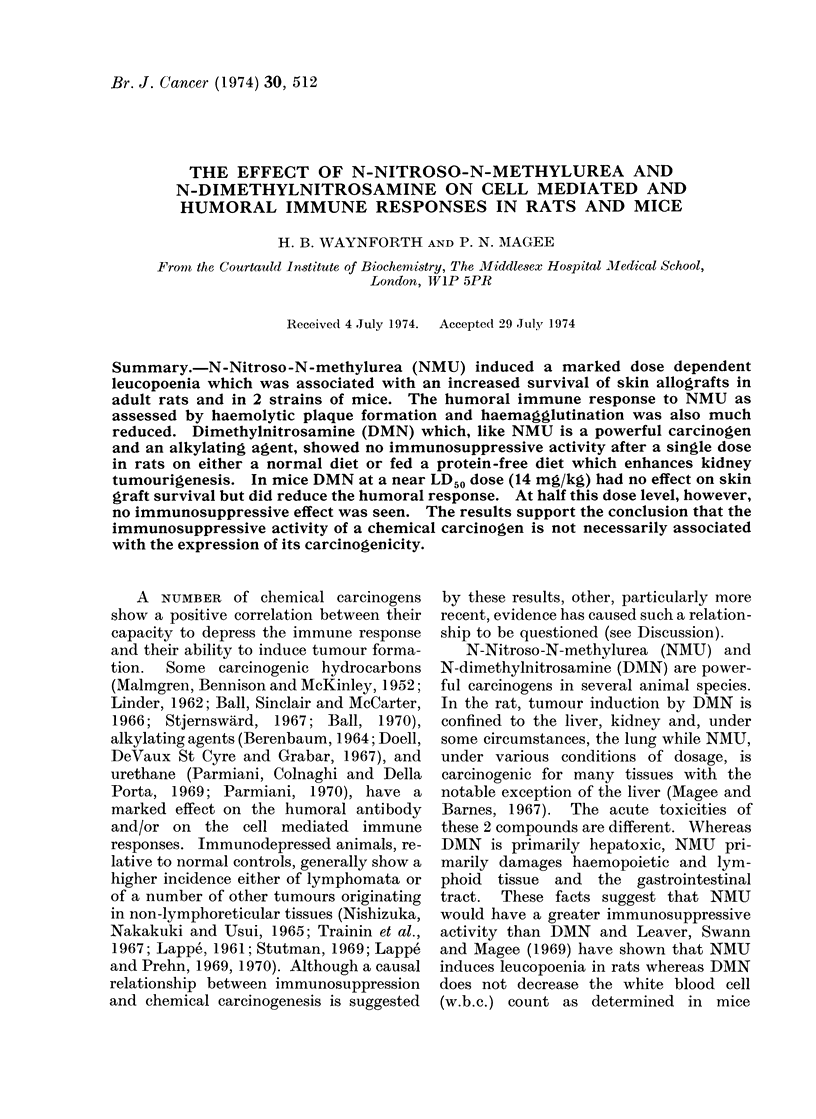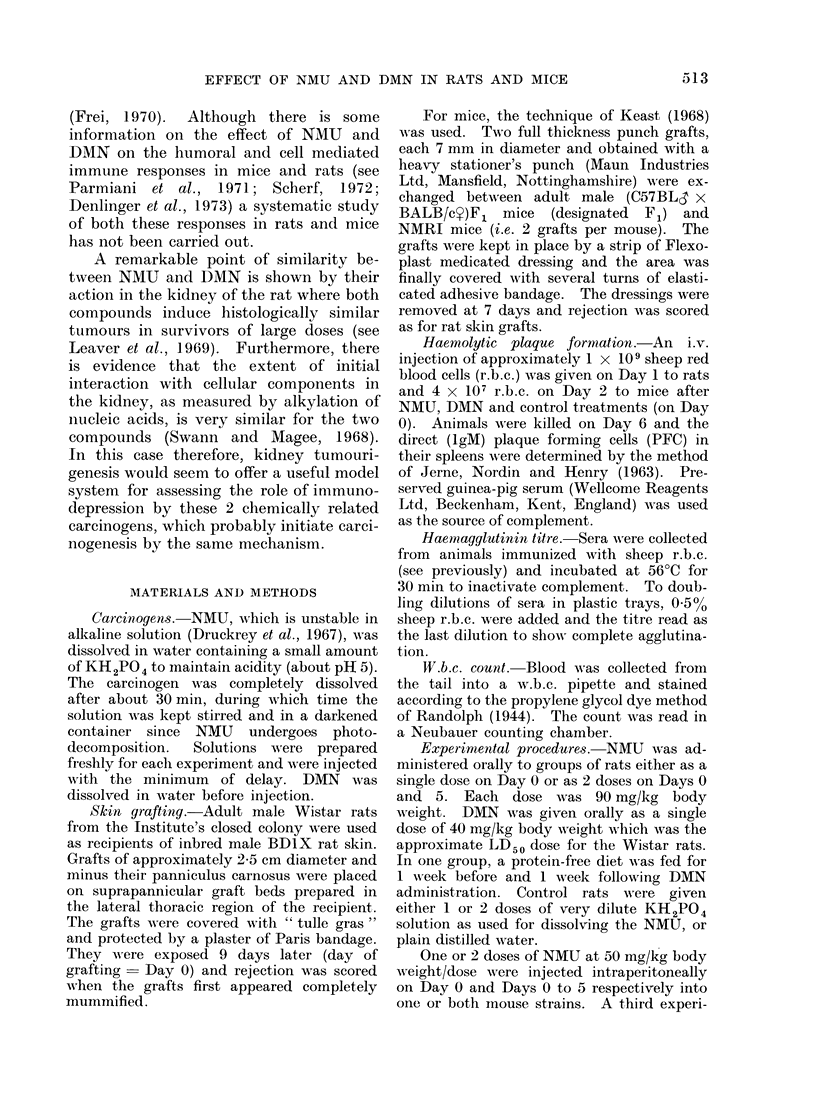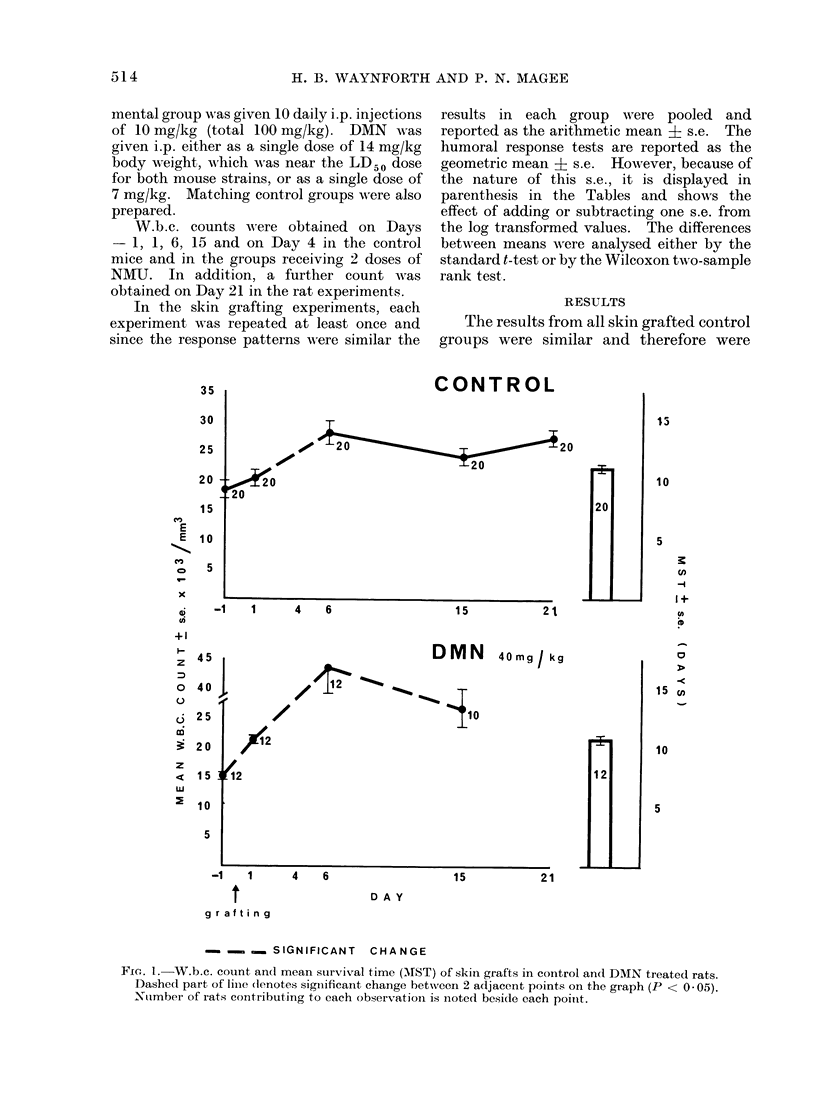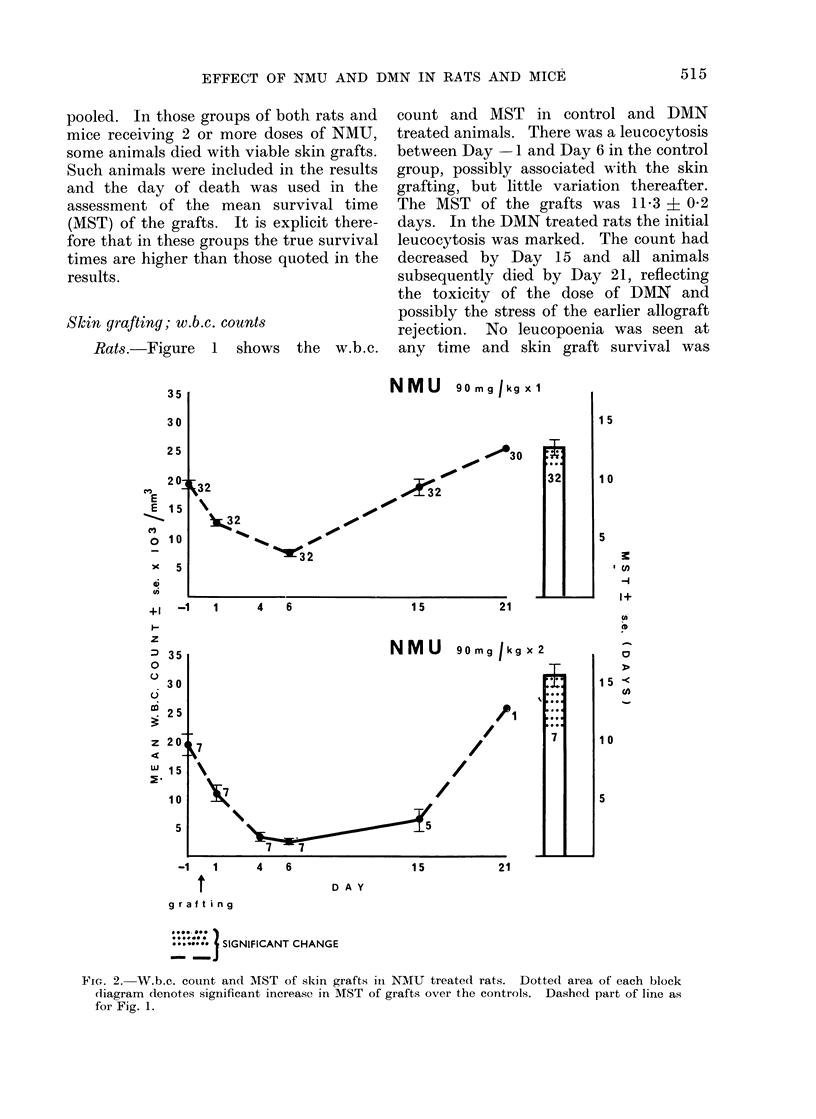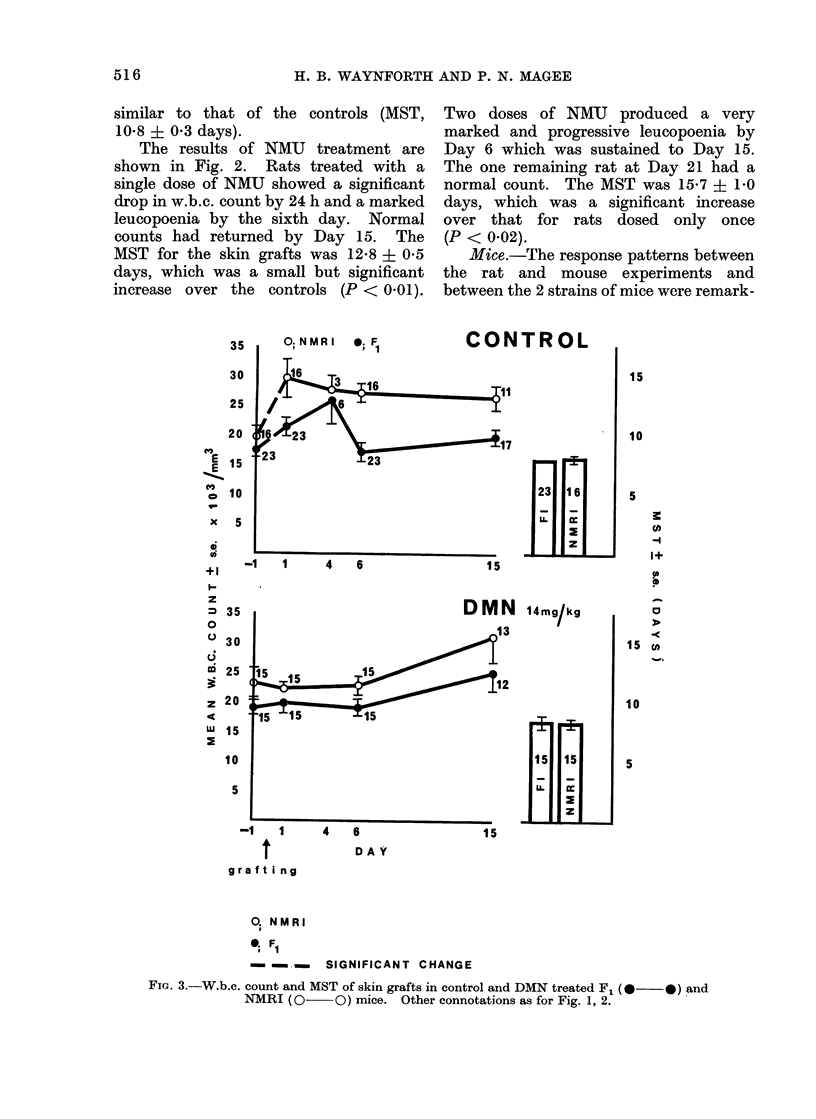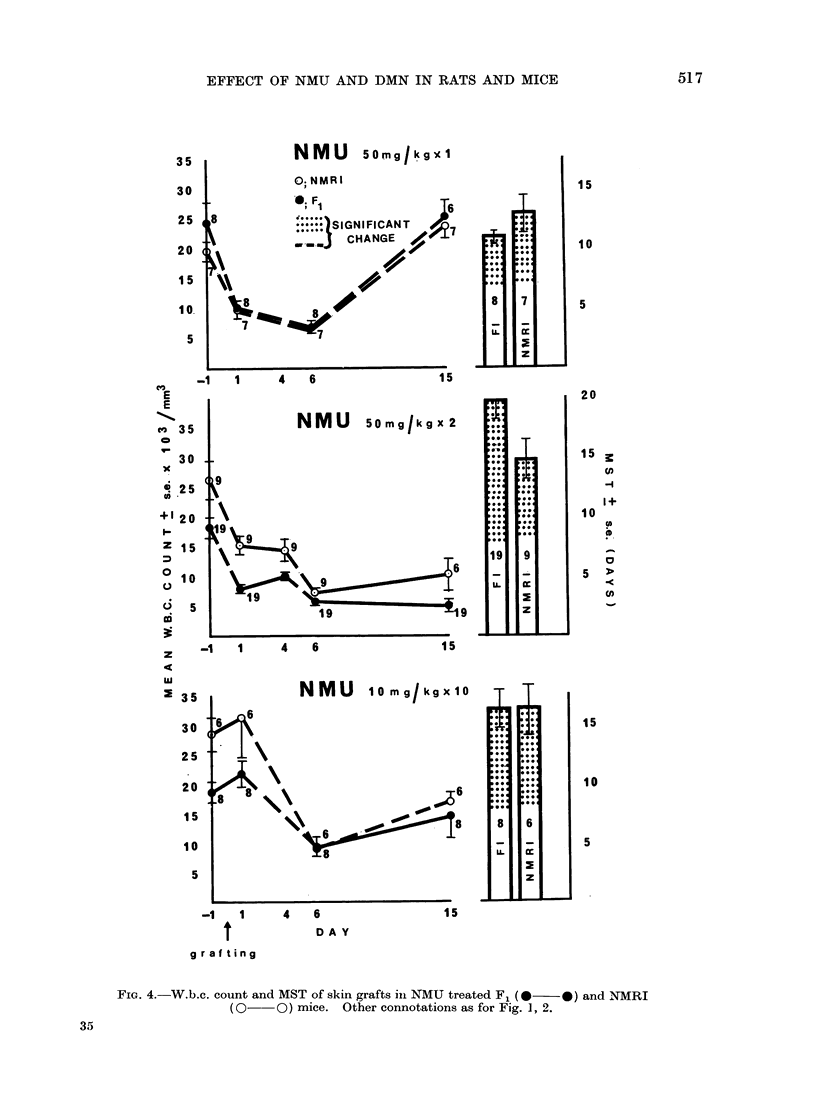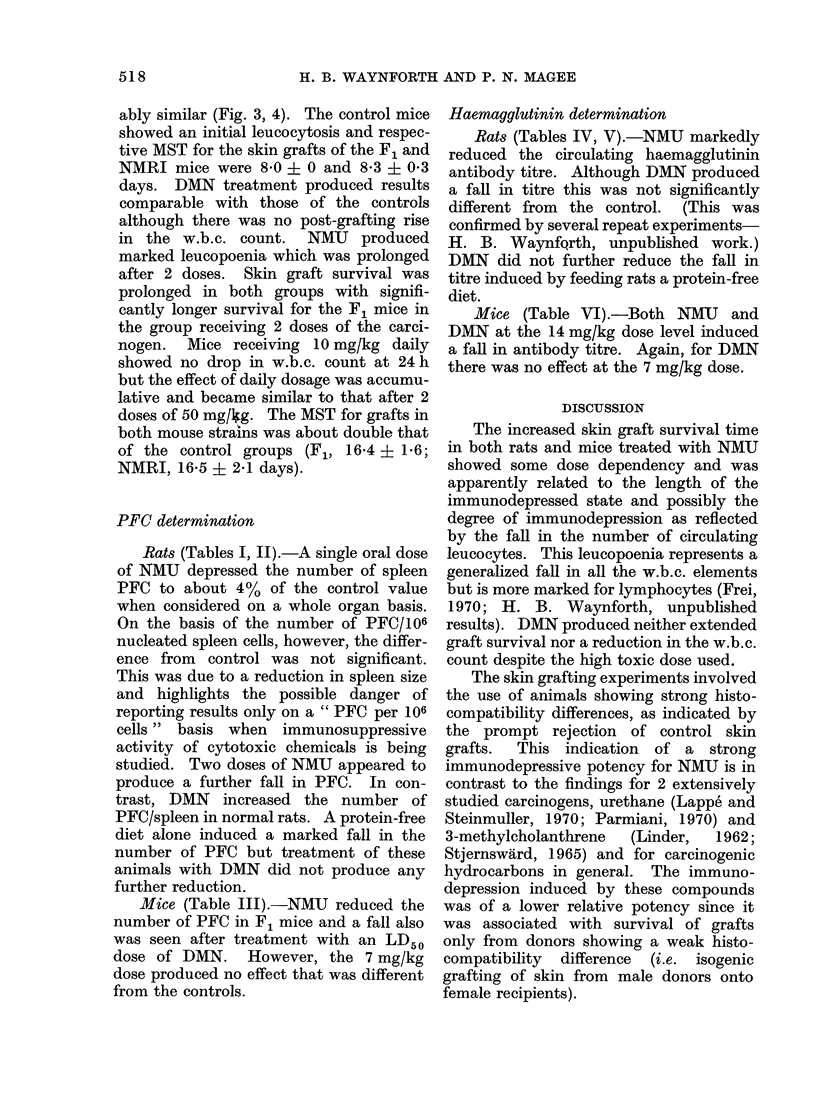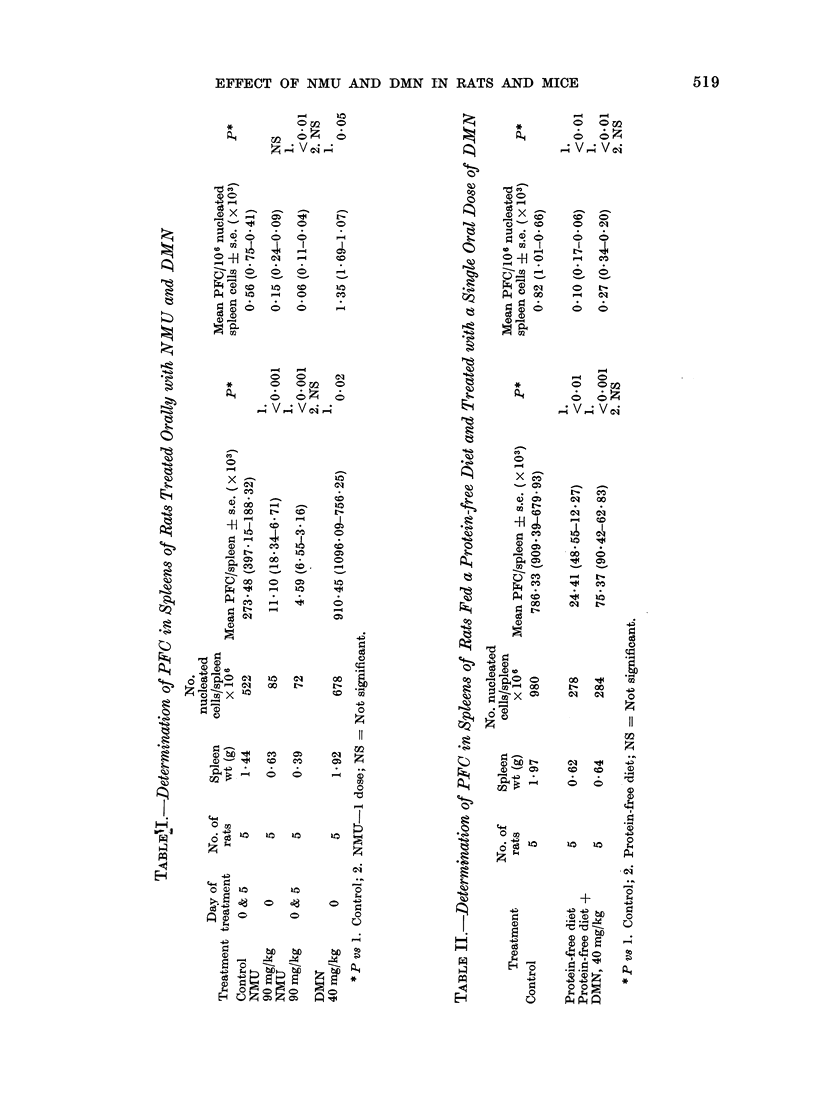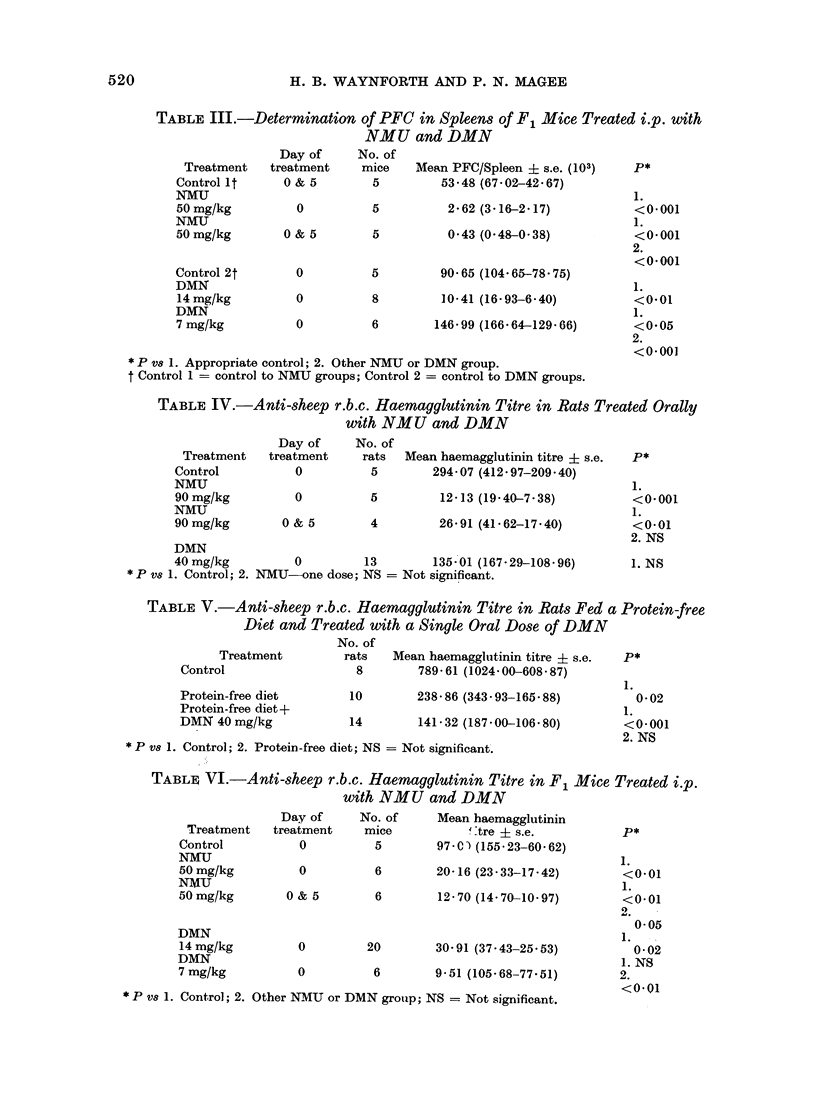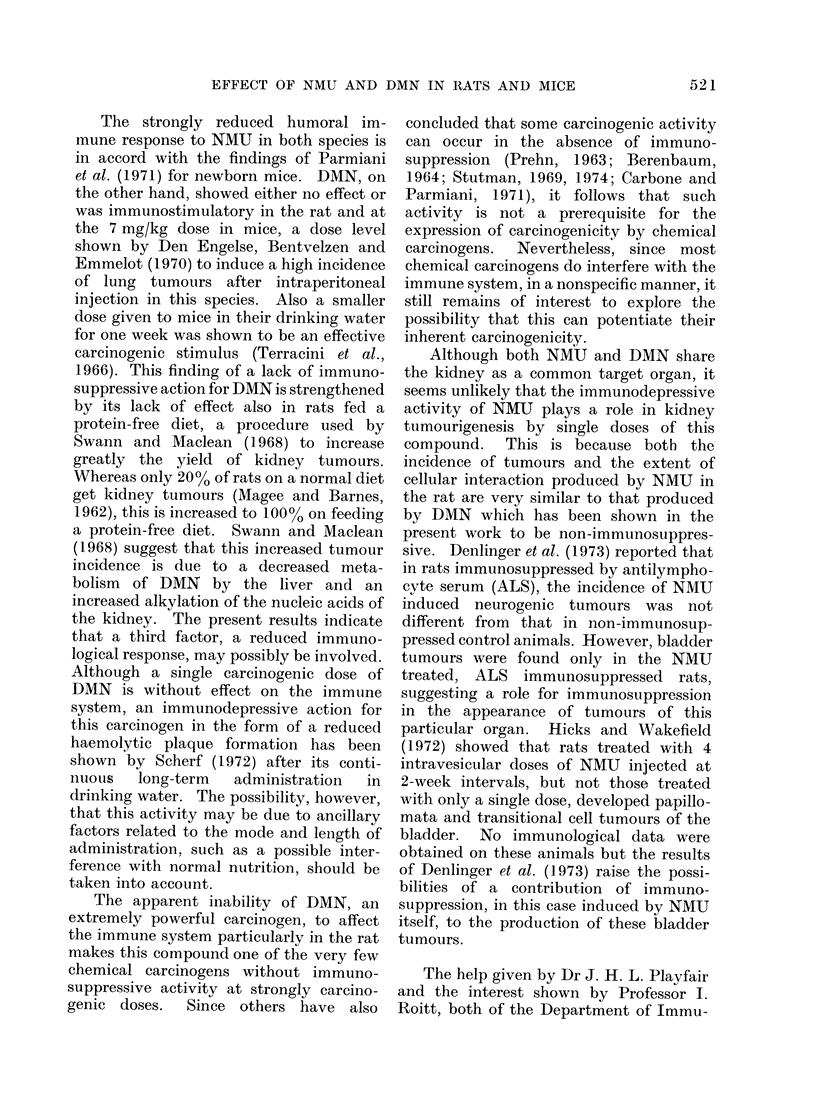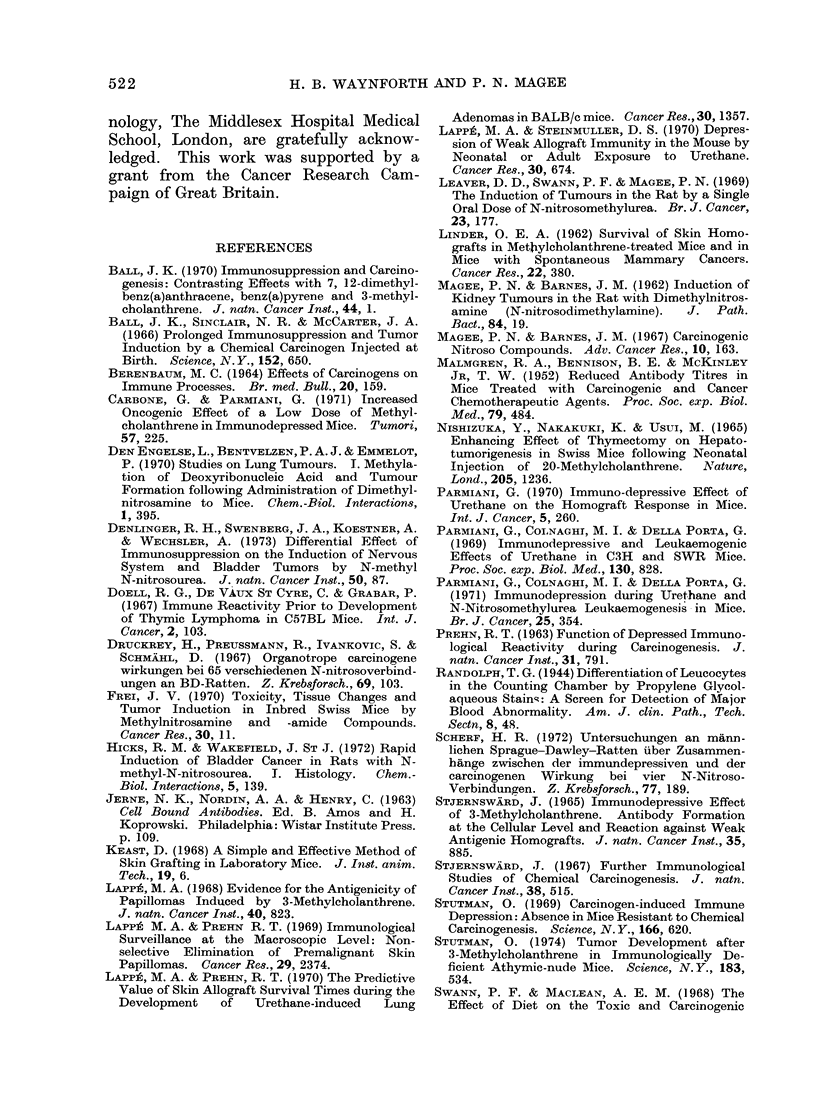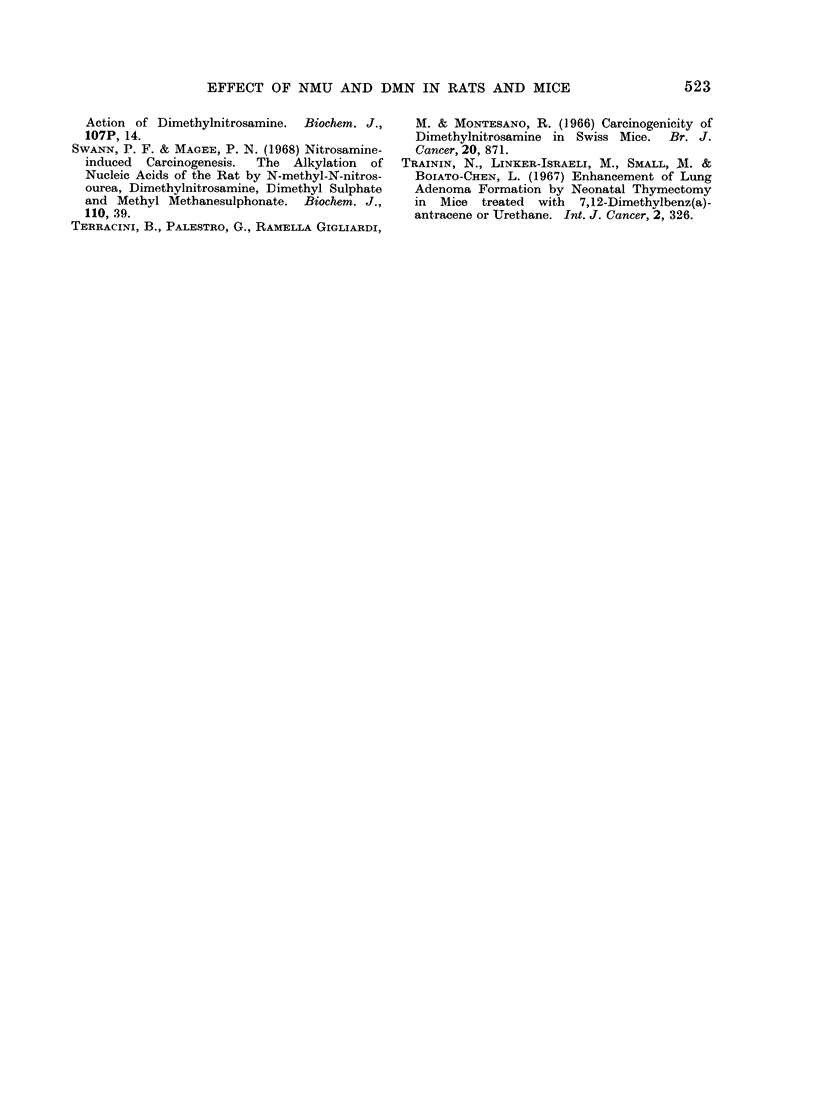# The Effect of N-Nitroso-N-Methylurea and N-Dimethylnitrosamine on Cell Mediated and Humoral Immune Responses in Rats and Mice

**DOI:** 10.1038/bjc.1974.230

**Published:** 1974-12

**Authors:** H. B. Waynforth, P. N. Magee

## Abstract

N-Nitroso-N-methylurea (NMU) induced a marked dose dependent leucopoenia which was associated with an increased survival of skin allografts in adult rats and in 2 strains of mice. The humoral immune response to NMU as assessed by haemolytic plaque formation and haemagglutination was also much reduced. Dimethylnitrosamine (DMN) which, like NMU is a powerful carcinogen and an alkylating agent, showed no immunosuppressive activity after a single dose in rats on either a normal diet or fed a protein-free diet which enhances kidney tumourigenesis. In mice DMN at a near LD_50_ dose (14 mg/kg) had no effect on skin graft survival but did reduce the humoral response. At half this dose level, however, no immunosuppressive effect was seen. The results support the conclusion that the immunosuppressive activity of a chemical carcinogen is not necessarily associated with the expression of its carcinogenicity.


Br. J. Cancer (1974) **30**, 512

### THE EFFECT OF N-NITROSO-N-METHYLUREA AND N-DIMETHYLNITROSAMINE ON CELL MEDIATED AND HUMORAL IMMUNE RESPONSES IN RATS AND MICE

H. B. WAYNFORTH AND P. N. MAGEE

*From the Courtauld Institute of Biochemistry, The Middlesex Hospital Medical School, London, W1P 5PR*

Received 4 July 1974. Accepted 29 July 1974

**Summary.—N-Nitroso-N-methylurea (NMU) induced a marked dose dependent leucopoenia which was associated with an increased survival of skin allografts in adult rats and in 2 strains of mice. The humoral immune response to NMU as assessed by haemolytic plaque formation and haemagglutination was also much reduced. Dimethylnitrosamine (DMN) which, like NMU is a powerful carcinogen and an alkylating agent, showed no immunosuppressive activity after a single dose in rats on either a normal diet or fed a protein-free diet which enhances kidney tumourigenesis. In mice DMN at a near $LD_{50}$ dose (14 mg/kg) had no effect on skin graft survival but did reduce the humoral response. At half this dose level, however, no immunosuppressive effect was seen. The results support the conclusion that the immunosuppressive activity of a chemical carcinogen is not necessarily associated with the expression of its carcinogenicity.**

A NUMBER of chemical carcinogens show a positive correlation between their capacity to depress the immune response and their ability to induce tumour formation. Some carcinogenic hydrocarbons (Malmgren, Bennison and McKinley, 1952; Linder, 1962; Ball, Sinclair and McCarter, 1966; Stjernswärd, 1967; Ball, 1970), alkylating agents (Berenbaum, 1964; Doell, DeVaux St Cyre and Grabar, 1967), and urethane (Parmiani, Colnaghi and Della Porta, 1969; Parmiani, 1970), have a marked effect on the humoral antibody and/or on the cell mediated immune responses. Immunodepressed animals, relative to normal controls, generally show a higher incidence either of lymphomata or of a number of other tumours originating in non-lymphoreticular tissues (Nishizuka, Nakakuki and Usui, 1965; Trainin *et al.*, 1967; Lappé, 1961; Stutman, 1969; Lappé and Prehn, 1969, 1970). Although a causal relationship between immunosuppression and chemical carcinogenesis is suggested by these results, other, particularly more recent, evidence has caused such a relationship to be questioned (see Discussion).

N-Nitroso-N-methylurea (NMU) and N-dimethylnitrosamine (DMN) are powerful carcinogens in several animal species. In the rat, tumour induction by DMN is confined to the liver, kidney and, under some circumstances, the lung while NMU, under various conditions of dosage, is carcinogenic for many tissues with the notable exception of the liver (Magee and Barnes, 1967). The acute toxicities of these 2 compounds are different. Whereas DMN is primarily hepatoxic, NMU primarily damages haemopoietic and lymphoid tissue and the gastrointestinal tract. These facts suggest that NMU would have a greater immunosuppressive activity than DMN and Leaver, Swann and Magee (1969) have shown that NMU induces leucopoenia in rats whereas DMN does not decrease the white blood cell (w.b.c.) count as determined in mice

(Frei, 1970). Although there is some information on the effect of NMU and DMN on the humoral and cell mediated immune responses in mice and rats (see Parmiani *et al.*, 1971; Scherf, 1972; Denlinger *et al.*, 1973) a systematic study of both these responses in rats and mice has not been carried out.

A remarkable point of similarity between NMU and DMN is shown by their action in the kidney of the rat where both compounds induce histologically similar tumours in survivors of large doses (see Leaver *et al.*, 1969). Furthermore, there is evidence that the extent of initial interaction with cellular components in the kidney, as measured by alkylation of nucleic acids, is very similar for the two compounds (Swann and Magee, 1968). In this case therefore, kidney tumourigenesis would seem to offer a useful model system for assessing the role of immunodepression by these 2 chemically related carcinogens, which probably initiate carcinogenesis by the same mechanism.

### MATERIALS AND METHODS

*Carcinogens.*—NMU, which is unstable in alkaline solution (Druckrey *et al.*, 1967), was dissolved in water containing a small amount of $KH_2PO_4$ to maintain acidity (about pH 5). The carcinogen was completely dissolved after about 30 min, during which time the solution was kept stirred and in a darkened container since NMU undergoes photodecomposition. Solutions were prepared freshly for each experiment and were injected with the minimum of delay. DMN was dissolved in water before injection.

*Skin grafting.*—Adult male Wistar rats from the Institute's closed colony were used as recipients of inbred male BD1X rat skin. Grafts of approximately 2·5 cm diameter and minus their panniculus carnosus were placed on suprapannicular graft beds prepared in the lateral thoracic region of the recipient. The grafts were covered with " tulle gras " and protected by a plaster of Paris bandage. They were exposed 9 days later (day of grafting = Day 0) and rejection was scored when the grafts first appeared completely mummified.

For mice, the technique of Keast (1968) was used. Two full thickness punch grafts, each 7 mm in diameter and obtained with a heavy stationer's punch (Maun Industries Ltd, Mansfield, Nottinghamshire) were exchanged between adult male (C57BL♂ × BALB/c♀)$F_1$ mice (designated $F_1$) and NMRI mice (*i.e.* 2 grafts per mouse). The grafts were kept in place by a strip of Flexoplast medicated dressing and the area was finally covered with several turns of elasticated adhesive bandage. The dressings were removed at 7 days and rejection was scored as for rat skin grafts.

*Haemolytic plaque formation.*—An i.v. injection of approximately $1 \times 10^9$ sheep red blood cells (r.b.c.) was given on Day 1 to rats and $4 \times 10^7$ r.b.c. on Day 2 to mice after NMU, DMN and control treatments (on Day 0). Animals were killed on Day 6 and the direct (IgM) plaque forming cells (PFC) in their spleens were determined by the method of Jerne, Nordin and Henry (1963). Preserved guinea-pig serum (Wellcome Reagents Ltd, Beckenham, Kent, England) was used as the source of complement.

*Haemagglutinin titre.*—Sera were collected from animals immunized with sheep r.b.c. (see previously) and incubated at 56°C for 30 min to inactivate complement. To doubling dilutions of sera in plastic trays, 0·5% sheep r.b.c. were added and the titre read as the last dilution to show complete agglutination.

*W.b.c. count.*—Blood was collected from the tail into a w.b.c. pipette and stained according to the propylene glycol dye method of Randolph (1944). The count was read in a Neubauer counting chamber.

*Experimental procedures.*—NMU was administered orally to groups of rats either as a single dose on Day 0 or as 2 doses on Days 0 and 5. Each dose was 90 mg/kg body weight. DMN was given orally as a single dose of 40 mg/kg body weight which was the approximate $LD_{50}$ dose for the Wistar rats. In one group, a protein-free diet was fed for 1 week before and 1 week following DMN administration. Control rats were given either 1 or 2 doses of very dilute $KH_2PO_4$ solution as used for dissolving the NMU, or plain distilled water.

One or 2 doses of NMU at 50 mg/kg body weight/dose were injected intraperitoneally on Day 0 and Days 0 to 5 respectively into one or both mouse strains. A third experimental group was given 10 daily i.p. injections of 10 mg/kg (total 100 mg/kg). DMN was given i.p. either as a single dose of 14 mg/kg body weight, which was near the $LD_{50}$ dose for both mouse strains, or as a single dose of 7 mg/kg. Matching control groups were also prepared.

W.b.c. counts were obtained on Days − 1, 1, 6, 15 and on Day 4 in the control mice and in the groups receiving 2 doses of NMU. In addition, a further count was obtained on Day 21 in the rat experiments.

In the skin grafting experiments, each experiment was repeated at least once and since the response patterns were similar the results in each group were pooled and reported as the arithmetic mean ± s.e. The humoral response tests are reported as the geometric mean ± s.e. However, because of the nature of this s.e., it is displayed in parenthesis in the Tables and shows the effect of adding or subtracting one s.e. from the log transformed values. The differences between means were analysed either by the standard $t$-test or by the Wilcoxon two-sample rank test.

### RESULTS

The results from all skin grafted control groups were similar and therefore were

Fig. 1.—W.b.c. count and mean survival time (MST) of skin grafts in control and DMN treated rats. Dashed part of line denotes significant change between 2 adjacent points on the graph ($P < 0{\cdot}05$). Number of rats contributing to each observation is noted beside each point.

pooled. In those groups of both rats and mice receiving 2 or more doses of NMU, some animals died with viable skin grafts. Such animals were included in the results and the day of death was used in the assessment of the mean survival time (MST) of the grafts. It is explicit therefore that in these groups the true survival times are higher than those quoted in the results.

*Skin grafting; w.b.c. counts*

*Rats.*—Figure 1 shows the w.b.c. count and MST in control and DMN treated animals. There was a leucocytosis between Day −1 and Day 6 in the control group, possibly associated with the skin grafting, but little variation thereafter. The MST of the grafts was 11·3 ± 0·2 days. In the DMN treated rats the initial leucocytosis was marked. The count had decreased by Day 15 and all animals subsequently died by Day 21, reflecting the toxicity of the dose of DMN and possibly the stress of the earlier allograft rejection. No leucopoenia was seen at any time and skin graft survival was

FIG. 2.—W.b.c. count and MST of skin grafts in NMU treated rats. Dotted area of each block diagram denotes significant increase in MST of grafts over the controls. Dashed part of line as for Fig. 1.

similar to that of the controls (MST, 10·8 ± 0·3 days).

The results of NMU treatment are shown in Fig. 2. Rats treated with a single dose of NMU showed a significant drop in w.b.c. count by 24 h and a marked leucopoenia by the sixth day. Normal counts had returned by Day 15. The MST for the skin grafts was 12·8 ± 0·5 days, which was a small but significant increase over the controls ($P < 0{\cdot}01$). Two doses of NMU produced a very marked and progressive leucopoenia by Day 6 which was sustained to Day 15. The one remaining rat at Day 21 had a normal count. The MST was 15·7 ± 1·0 days, which was a significant increase over that for rats dosed only once ($P < 0{\cdot}02$).

*Mice.*—The response patterns between the rat and mouse experiments and between the 2 strains of mice were remark-

FIG. 3.—W.b.c. count and MST of skin grafts in control and DMN treated $F_1$ (●——●) and NMRI (○——○) mice. Other connotations as for Fig. 1, 2.

FIG. 4.—W.b.c. count and MST of skin grafts in NMU treated $F_1$ (●——●) and NMRI (○——○) mice. Other connotations as for Fig. 1, 2.

ably similar (Fig. 3, 4). The control mice showed an initial leucocytosis and respective MST for the skin grafts of the $F_1$ and NMRI mice were $8{\cdot}0 \pm 0$ and $8{\cdot}3 \pm 0{\cdot}3$ days. DMN treatment produced results comparable with those of the controls although there was no post-grafting rise in the w.b.c. count. NMU produced marked leucopoenia which was prolonged after 2 doses. Skin graft survival was prolonged in both groups with significantly longer survival for the $F_1$ mice in the group receiving 2 doses of the carcinogen. Mice receiving 10 mg/kg daily showed no drop in w.b.c. count at 24 h but the effect of daily dosage was accumulative and became similar to that after 2 doses of 50 mg/kg. The MST for grafts in both mouse strains was about double that of the control groups ($F_1$, $16{\cdot}4 \pm 1{\cdot}6$; NMRI, $16{\cdot}5 \pm 2{\cdot}1$ days).

*PFC determination*

*Rats* (Tables I, II).—A single oral dose of NMU depressed the number of spleen PFC to about 4% of the control value when considered on a whole organ basis. On the basis of the number of PFC/$10^6$ nucleated spleen cells, however, the difference from control was not significant. This was due to a reduction in spleen size and highlights the possible danger of reporting results only on a " PFC per $10^6$ cells " basis when immunosuppressive activity of cytotoxic chemicals is being studied. Two doses of NMU appeared to produce a further fall in PFC. In contrast, DMN increased the number of PFC/spleen in normal rats. A protein-free diet alone induced a marked fall in the number of PFC but treatment of these animals with DMN did not produce any further reduction.

*Mice* (Table III).—NMU reduced the number of PFC in $F_1$ mice and a fall also was seen after treatment with an $LD_{50}$ dose of DMN. However, the 7 mg/kg dose produced no effect that was different from the controls.

*Haemagglutinin determination*

*Rats* (Tables IV, V).—NMU markedly reduced the circulating haemagglutinin antibody titre. Although DMN produced a fall in titre this was not significantly different from the control. (This was confirmed by several repeat experiments—H. B. Waynforth, unpublished work.) DMN did not further reduce the fall in titre induced by feeding rats a protein-free diet.

*Mice* (Table VI).—Both NMU and DMN at the 14 mg/kg dose level induced a fall in antibody titre. Again, for DMN there was no effect at the 7 mg/kg dose.

### DISCUSSION

The increased skin graft survival time in both rats and mice treated with NMU showed some dose dependency and was apparently related to the length of the immunodepressed state and possibly the degree of immunodepression as reflected by the fall in the number of circulating leucocytes. This leucopoenia represents a generalized fall in all the w.b.c. elements but is more marked for lymphocytes (Frei, 1970; H. B. Waynforth, unpublished results). DMN produced neither extended graft survival nor a reduction in the w.b.c. count despite the high toxic dose used.

The skin grafting experiments involved the use of animals showing strong histocompatibility differences, as indicated by the prompt rejection of control skin grafts. This indication of a strong immunodepressive potency for NMU is in contrast to the findings for 2 extensively studied carcinogens, urethane (Lappé and Steinmuller, 1970; Parmiani, 1970) and 3-methylcholanthrene (Linder, 1962; Stjernswärd, 1965) and for carcinogenic hydrocarbons in general. The immunodepression induced by these compounds was of a lower relative potency since it was associated with survival of grafts only from donors showing a weak histocompatibility difference (*i.e.* isogenic grafting of skin from male donors onto female recipients).

TABLE I.—*Determination of PFC in Spleens of Rats Treated Orally with NMU and DMN*

| Treatment | Day of treatment | No. of rats | Spleen wt (g) | No. nucleated cells/spleen × 10⁶ | Mean PFC/spleen ± s.e. (× 10³) | P* | Mean PFC/10⁶ nucleated spleen cells ± s.e. (× 10³) | P* |
|---|---|---|---|---|---|---|---|---|
| Control | 0 & 5 | 5 | 1·44 | 522 | 273·48 (397·15–188·32) | | 0·56 (0·75–0·41) | |
| NMU 90 mg/kg | 0 | 5 | 0·63 | 85 | 11·10 (18·34–6·71) | 1. <0·001 | 0·15 (0·24–0·09) | NS |
| NMU 90 mg/kg | 0 & 5 | 5 | 0·39 | 72 | 4·59 (6·55–3·16) | 1. <0·001 2. NS | 0·06 (0·11–0·04) | 1. <0·01 2. NS |
| DMN 40 mg/kg | 0 | 5 | 1·92 | 678 | 910·45 (1096·09–756·25) | 1. 0·02 | 1·35 (1·69–1·07) | 1. 0·05 |

* $P$ *vs* 1. Control; 2. NMU—1 dose; NS = Not significant.

TABLE II.—*Determination of PFC in Spleens of Rats Fed a Protein-free Diet and Treated with a Single Oral Dose of DMN*

| Treatment | No. of rats | Spleen wt (g) | No. nucleated cells/spleen × 10⁶ | Mean PFC/spleen ± s.e. (× 10³) | P* | Mean PFC/10⁶ nucleated spleen cells ± s.e. (× 10³) | P* |
|---|---|---|---|---|---|---|---|
| Control | 5 | 1·97 | 980 | 786·33 (909·39–679·93) | | 0·82 (1·01–0·66) | |
| Protein-free diet | 5 | 0·62 | 278 | 24·41 (48·55–12·27) | 1. <0·01 | 0·10 (0·17–0·06) | 1. <0·01 |
| Protein-free diet + DMN, 40 mg/kg | 5 | 0·64 | 284 | 75·37 (90·42–62·83) | 1. <0·001 2. NS | 0·27 (0·34–0·20) | 1. <0·01 2. NS |

* $P$ *vs* 1. Control; 2. Protein-free diet; NS = Not significant.

TABLE III.—*Determination of PFC in Spleens of $F_1$ Mice Treated i.p. with NMU and DMN*

| Treatment | Day of treatment | No. of mice | Mean PFC/Spleen ± s.e. ($10^3$) | P* |
|---|---|---|---|---|
| Control 1† | 0 & 5 | 5 | 53·48 (67·02–42·67) | |
| NMU 50 mg/kg | 0 | 5 | 2·62 (3·16–2·17) | 1. <0·001 |
| NMU 50 mg/kg | 0 & 5 | 5 | 0·43 (0·48–0·38) | 1. <0·001 2. <0·001 |
| Control 2† | 0 | 5 | 90·65 (104·65–78·75) | |
| DMN 14 mg/kg | 0 | 8 | 10·41 (16·93–6·40) | 1. <0·01 |
| DMN 7 mg/kg | 0 | 6 | 146·99 (166·64–129·66) | 1. <0·05 2. <0·001 |

* *P vs* 1. Appropriate control; 2. Other NMU or DMN group.
† Control 1 = control to NMU groups; Control 2 = control to DMN groups.

TABLE IV.—*Anti-sheep r.b.c. Haemagglutinin Titre in Rats Treated Orally with NMU and DMN*

| Treatment | Day of treatment | No. of rats | Mean haemagglutinin titre ± s.e. | P* |
|---|---|---|---|---|
| Control | 0 | 5 | 294·07 (412·97–209·40) | |
| NMU 90 mg/kg | 0 | 5 | 12·13 (19·40–7·38) | 1. <0·001 |
| NMU 90 mg/kg | 0 & 5 | 4 | 26·91 (41·62–17·40) | 1. <0·01 2. NS |
| DMN 40 mg/kg | 0 | 13 | 135·01 (167·29–108·96) | 1. NS |

* *P vs* 1. Control; 2. NMU—one dose; NS = Not significant.

TABLE V.—*Anti-sheep r.b.c. Haemagglutinin Titre in Rats Fed a Protein-free Diet and Treated with a Single Oral Dose of DMN*

| Treatment | No. of rats | Mean haemagglutinin titre ± s.e. | P* |
|---|---|---|---|
| Control | 8 | 789·61 (1024·00–608·87) | |
| Protein-free diet | 10 | 238·86 (343·93–165·88) | 1. 0·02 |
| Protein-free diet + DMN 40 mg/kg | 14 | 141·32 (187·00–106·80) | 1. <0·001 2. NS |

* *P vs* 1. Control; 2. Protein-free diet; NS = Not significant.

TABLE VI.—*Anti-sheep r.b.c. Haemagglutinin Titre in $F_1$ Mice Treated i.p. with NMU and DMN*

| Treatment | Day of treatment | No. of mice | Mean haemagglutinin titre ± s.e. | P* |
|---|---|---|---|---|
| Control | 0 | 5 | 97·0[illegible] (155·23–60·62) | |
| NMU 50 mg/kg | 0 | 6 | 20·16 (23·33–17·42) | 1. <0·01 |
| NMU 50 mg/kg | 0 & 5 | 6 | 12·70 (14·70–10·97) | 1. <0·01 2. 0·05 |
| DMN 14 mg/kg | 0 | 20 | 30·91 (37·43–25·53) | 1. 0·02 |
| DMN 7 mg/kg | 0 | 6 | 9·51 (105·68–77·51) | 1. NS 2. <0·01 |

* *P vs* 1. Control; 2. Other NMU or DMN group; NS = Not significant.

The strongly reduced humoral immune response to NMU in both species is in accord with the findings of Parmiani *et al.* (1971) for newborn mice. DMN, on the other hand, showed either no effect or was immunostimulatory in the rat and at the 7 mg/kg dose in mice, a dose level shown by Den Engelse, Bentvelzen and Emmelot (1970) to induce a high incidence of lung tumours after intraperitoneal injection in this species. Also a smaller dose given to mice in their drinking water for one week was shown to be an effective carcinogenic stimulus (Terracini *et al.*, 1966). This finding of a lack of immunosuppressive action for DMN is strengthened by its lack of effect also in rats fed a protein-free diet, a procedure used by Swann and Maclean (1968) to increase greatly the yield of kidney tumours. Whereas only 20% of rats on a normal diet get kidney tumours (Magee and Barnes, 1962), this is increased to 100% on feeding a protein-free diet. Swann and Maclean (1968) suggest that this increased tumour incidence is due to a decreased metabolism of DMN by the liver and an increased alkylation of the nucleic acids of the kidney. The present results indicate that a third factor, a reduced immunological response, may possibly be involved. Although a single carcinogenic dose of DMN is without effect on the immune system, an immunodepressive action for this carcinogen in the form of a reduced haemolytic plaque formation has been shown by Scherf (1972) after its continuous long-term administration in drinking water. The possibility, however, that this activity may be due to ancillary factors related to the mode and length of administration, such as a possible interference with normal nutrition, should be taken into account.

The apparent inability of DMN, an extremely powerful carcinogen, to affect the immune system particularly in the rat makes this compound one of the very few chemical carcinogens without immunosuppressive activity at strongly carcinogenic doses. Since others have also concluded that some carcinogenic activity can occur in the absence of immunosuppression (Prehn, 1963; Berenbaum, 1964; Stutman, 1969, 1974; Carbone and Parmiani, 1971), it follows that such activity is not a prerequisite for the expression of carcinogenicity by chemical carcinogens. Nevertheless, since most chemical carcinogens do interfere with the immune system, in a nonspecific manner, it still remains of interest to explore the possibility that this can potentiate their inherent carcinogenicity.

Although both NMU and DMN share the kidney as a common target organ, it seems unlikely that the immunodepressive activity of NMU plays a role in kidney tumourigenesis by single doses of this compound. This is because both the incidence of tumours and the extent of cellular interaction produced by NMU in the rat are very similar to that produced by DMN which has been shown in the present work to be non-immunosuppressive. Denlinger *et al.* (1973) reported that in rats immunosuppressed by antilymphocyte serum (ALS), the incidence of NMU induced neurogenic tumours was not different from that in non-immunosuppressed control animals. However, bladder tumours were found only in the NMU treated, ALS immunosuppressed rats, suggesting a role for immunosuppression in the appearance of tumours of this particular organ. Hicks and Wakefield (1972) showed that rats treated with 4 intravesicular doses of NMU injected at 2-week intervals, but not those treated with only a single dose, developed papillomata and transitional cell tumours of the bladder. No immunological data were obtained on these animals but the results of Denlinger *et al.* (1973) raise the possibilities of a contribution of immunosuppression, in this case induced by NMU itself, to the production of these bladder tumours.

The help given by Dr J. H. L. Playfair and the interest shown by Professor I. Roitt, both of the Department of Immunology, The Middlesex Hospital Medical School, London, are gratefully acknowledged. This work was supported by a grant from the Cancer Research Campaign of Great Britain.

### REFERENCES

BALL, J. K. (1970) Immunosuppression and Carcinogenesis: Contrasting Effects with 7, 12-dimethylbenz(a)anthracene, benz(a)pyrene and 3-methylcholanthrene. *J. natn. Cancer Inst.*, **44**, 1.

BALL, J. K., SINCLAIR, N. R. & MCCARTER, J. A. (1966) Prolonged Immunosuppression and Tumor Induction by a Chemical Carcinogen Injected at Birth. *Science, N.Y.*, **152**, 650.

BERENBAUM, M. C. (1964) Effects of Carcinogens on Immune Processes. *Br. med. Bull.*, **20**, 159.

CARBONE, G. & PARMIANI, G. (1971) Increased Oncogenic Effect of a Low Dose of Methylcholanthrene in Immunodepressed Mice. *Tumori*, **57**, 225.

DEN ENGELSE, L., BENTVELZEN, P. A. J. & EMMELOT, P. (1970) Studies on Lung Tumours. I. Methylation of Deoxyribonucleic Acid and Tumour Formation following Administration of Dimethylnitrosamine to Mice. *Chem.-Biol. Interactions*, **1**, 395.

DENLINGER, R. H., SWENBERG, J. A., KOESTNER, A. & WECHSLER, A. (1973) Differential Effect of Immunosuppression on the Induction of Nervous System and Bladder Tumors by N-methyl N-nitrosourea. *J. natn. Cancer Inst.*, **50**, 87.

DOELL, R. G., DE VAUX ST CYRE, C. & GRABAR, P. (1967) Immune Reactivity Prior to Development of Thymic Lymphoma in C57BL Mice. *Int. J. Cancer*, **2**, 103.

DRUCKREY, H., PREUSSMANN, R., IVANKOVIC, S. & SCHMÄHL, D. (1967) Organotrope carcinogene wirkungen bei 65 verschiedenen N-nitrosoverbindungen an BD-Ratten. *Z. Krebsforsch.*, **69**, 103.

FREI, J. V. (1970) Toxicity, Tissue Changes and Tumor Induction in Inbred Swiss Mice by Methylnitrosamine and -amide Compounds. *Cancer Res.*, **30**, 11.

HICKS, R. M. & WAKEFIELD, J. ST J. (1972) Rapid Induction of Bladder Cancer in Rats with N-methyl-N-nitrosourea. I. Histology. *Chem.-Biol. Interactions*, **5**, 139.

JERNE, N. K., NORDIN, A. A. & HENRY, C. (1963) *Cell Bound Antibodies*. Ed. B. Amos and H. Koprowski. Philadelphia: Wistar Institute Press. p. 109.

KEAST, D. (1968) A Simple and Effective Method of Skin Grafting in Laboratory Mice. *J. Inst. anim. Tech.*, **19**, 6.

LAPPÉ, M. A. (1968) Evidence for the Antigenicity of Papillomas Induced by 3-Methylcholanthrene. *J. natn. Cancer Inst.*, **40**, 823.

LAPPÉ M. A. & PREHN R. T. (1969) Immunological Surveillance at the Macroscopic Level: Nonselective Elimination of Premalignant Skin Papillomas. *Cancer Res.*, **29**, 2374.

LAPPÉ, M. A. & PREHN, R. T. (1970) The Predictive Value of Skin Allograft Survival Times during the Development of Urethane-induced Lung Adenomas in BALB/c mice. *Cancer Res.*, **30**, 1357.

LAPPÉ, M. A. & STEINMULLER, D. S. (1970) Depression of Weak Allograft Immunity in the Mouse by Neonatal or Adult Exposure to Urethane. *Cancer Res.*, **30**, 674.

LEAVER, D. D., SWANN, P. F. & MAGEE, P. N. (1969) The Induction of Tumours in the Rat by a Single Oral Dose of N-nitrosomethylurea. *Br. J. Cancer*, **23**, 177.

LINDER, O. E. A. (1962) Survival of Skin Homografts in Methylcholanthrene-treated Mice and in Mice with Spontaneous Mammary Cancers. *Cancer Res.*, **22**, 380.

MAGEE, P. N. & BARNES, J. M. (1962) Induction of Kidney Tumours in the Rat with Dimethylnitrosamine (N-nitrosodimethylamine). *J. Path. Bact.*, **84**, 19.

MAGEE, P. N. & BARNES, J. M. (1967) Carcinogenic Nitroso Compounds. *Adv. Cancer Res.*, **10**, 163.

MALMGREN, R. A., BENNISON, B. E. & MCKINLEY JR, T. W. (1952) Reduced Antibody Titres in Mice Treated with Carcinogenic and Cancer Chemotherapeutic Agents. *Proc. Soc. exp. Biol. Med.*, **79**, 484.

NISHIZUKA, Y., NAKAKUKI, K. & USUI, M. (1965) Enhancing Effect of Thymectomy on Hepatotumorigenesis in Swiss Mice following Neonatal Injection of 20-Methylcholanthrene. *Nature, Lond.*, **205**, 1236.

PARMIANI, G. (1970) Immuno-depressive Effect of Urethane on the Homograft Response in Mice. *Int. J. Cancer*, **5**, 260.

PARMIANI, G., COLNAGHI, M. I. & DELLA PORTA, G. (1969) Immunodepressive and Leukaemogenic Effects of Urethane in C3H and SWR Mice. *Proc. Soc. exp. Biol. Med.*, **130**, 828.

PARMIANI, G., COLNAGHI, M. I. & DELLA PORTA, G. (1971) Immunodepression during Urethane and N-Nitrosomethylurea Leukaemogenesis in Mice. *Br. J. Cancer*, **25**, 354.

PREHN, R. T. (1963) Function of Depressed Immunological Reactivity during Carcinogenesis. *J. natn. Cancer Inst.*, **31**, 791.

RANDOLPH, T. G. (1944) Differentiation of Leucocytes in the Counting Chamber by Propylene Glycol-aqueous Stains: A Screen for Detection of Major Blood Abnormality. *Am. J. clin. Path., Tech. Sectn*, **8**, 48.

SCHERF, H. R. (1972) Untersuchungen an männlichen Sprague–Dawley–Ratten über Zusammenhänge zwischen der immundepressiven und der carcinogenen Wirkung bei vier N-Nitroso-Verbindungen. *Z. Krebsforsch.*, **77**, 189.

STJERNSWÄRD, J. (1965) Immunodepressive Effect of 3-Methylcholanthrene. Antibody Formation at the Cellular Level and Reaction against Weak Antigenic Homografts. *J. natn. Cancer Inst.*, **35**, 885.

STJERNSWÄRD, J. (1967) Further Immunological Studies of Chemical Carcinogenesis. *J. natn. Cancer Inst.*, **38**, 515.

STUTMAN, O. (1969) Carcinogen-induced Immune Depression: Absence in Mice Resistant to Chemical Carcinogenesis. *Science, N.Y.*, **166**, 620.

STUTMAN, O. (1974) Tumor Development after 3-Methylcholanthrene in Immunologically Deficient Athymic-nude Mice. *Science, N.Y.*, **183**, 534.

SWANN, P. F. & MACLEAN, A. E. M. (1968) The Effect of Diet on the Toxic and Carcinogenic

Action of Dimethylnitrosamine. *Biochem. J.*, **107P**, 14.
SWANN, P. F. & MAGEE, P. N. (1968) Nitrosamine-induced Carcinogenesis. The Alkylation of Nucleic Acids of the Rat by N-methyl-N-nitrosourea, Dimethylnitrosamine, Dimethyl Sulphate and Methyl Methanesulphonate. *Biochem. J.*, **110**, 39.
TERRACINI, B., PALESTRO, G., RAMELLA GIGLIARDI, M. & MONTESANO, R. (1966) Carcinogenicity of Dimethylnitrosamine in Swiss Mice. *Br. J. Cancer*, **20**, 871.
TRAININ, N., LINKER-ISRAELI, M., SMALL, M. & BOIATO-CHEN, L. (1967) Enhancement of Lung Adenoma Formation by Neonatal Thymectomy in Mice treated with 7,12-Dimethylbenz(a)-antracene or Urethane. *Int. J. Cancer*, **2**, 326.